# Post-translational modifications of transthyretin affect the triiodonine-binding potential

**DOI:** 10.1111/jcmm.12446

**Published:** 2014-10-14

**Authors:** Andrea Henze, Thomas Homann, Mustafa Serteser, Ozge Can, Ozlem Sezgin, Abdurrahman Coskun, Ibrahim Unsal, Florian J Schweigert, Aysel Ozpinar

**Affiliations:** a Institute of Nutrition, University of PotsdamNuthetal, Germany; b Department of Medical Biochemistry and Internal Medicine, Acibadem UniversityIstanbul, Turkey

**Keywords:** transthyretin, triiodthyronine, cysteine oxidation, post-translational modifications, thyroid hormones, molecular modelling, mass spectrometry

## Abstract

Transthyretin (TTR) is a visceral protein, which facilitates the transport of thyroid hormones in blood and cerebrospinal fluid. The homotetrameric structure of TTR enables the simultaneous binding of two thyroid hormones per molecule. Each TTR subunit provides a single cysteine residue (Cys_10_), which is frequently affected by oxidative post-translational modifications. As Cys_10_ is part of the thyroid hormone-binding channel within the TTR molecule, PTM of Cys_10_ may influence the binding of thyroid hormones. Therefore, we analysed the effects of Cys_10_ modification with sulphonic acid, cysteine, cysteinylglycine and glutathione on binding of triiodothyronine (T3) by molecular modelling. Furthermore, we determined the PTM pattern of TTR in serum of patients with thyroid disease by immunoprecipitation and mass spectrometry to evaluate this association *in vivo*. The *in silico* assays demonstrated that oxidative PTM of TTR resulted in substantial reorganization of the intramolecular interactions and also affected the binding of T3 in a chemotype- and site-specific manner with S-glutathionylation as the most potent modulator of T3 binding. These findings were supported by the *in vivo* results, which indicated thyroid function-specific patterns of TTR with a substantial decrease in S-sulphonated, S-cysteinylglycinated and S-glutathionylated TTR in hypothyroid patients. In conclusion, this study provides evidence that oxidative modifications of Cys_10_ seem to affect binding of T3 to TTR probably because of the introduction of a sterical hindrance and induction of conformational changes. As oxidative modifications can be dynamically regulated, this may represent a sensitive mechanism to adjust thyroid hormone availability.

## Introduction

**Fig 1:**
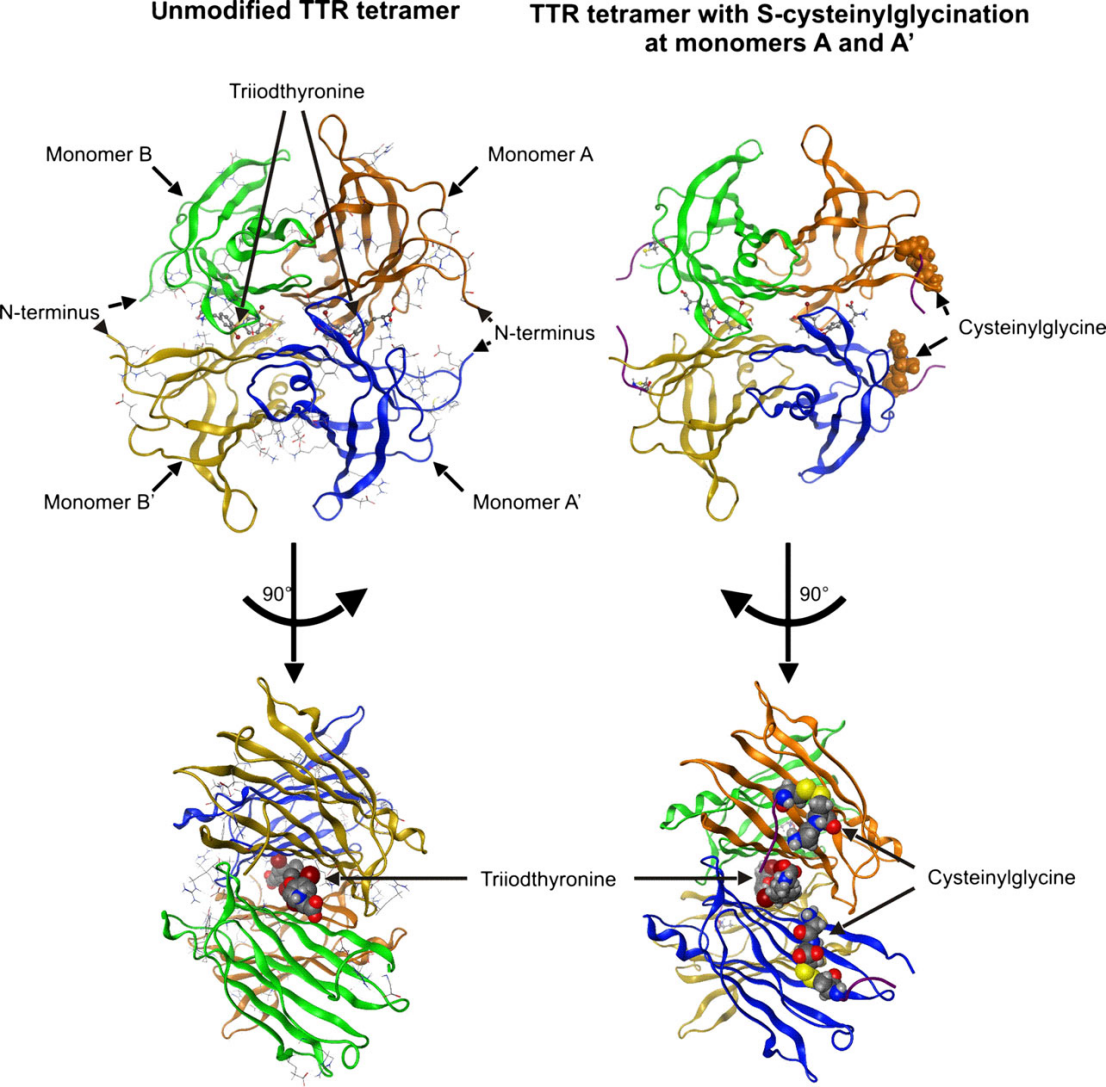
Ribbon models of the transthyretin–triiodothyronine complex with unmodified Cys_10_ and Cys_10_ modified by S-cysteinylglycination in monomers A and A'. TTR is a tetramer composed of four identical subunits, which are annotated as monomer A (orange), monomer A' (blue), monomer B (green) and monomer B' (yellow). Monomers A and B associate to form a dimer, as do the monomers A' and B'. Subsequently, both dimers associate to form the tetramer. The thyroid hormone-binding cavities are located at the interface between the dimer units and their entrances are formed by the monomers AA' and BB' respectively 2,3.

Transthyretin (TTR) is a visceral protein, which is mainly synthesized in the liver, but also in extrahepatic tissues such as the brain and the pancreas 1. Among other things, TTR is responsible for the transport of lipophilic substances in the body and this is facilitated by the exceptional organization of the TTR molecule. TTR is composed of 127 amino acids, which are arranged in a β-sheet-rich structure. Two TTR subunits (annotated in this paper as monomer A and monomer B) dimerize and subsequently two dimers (dimer AB and dimer A'B') associate to form a tetramer (Fig.1A), which is finally secreted 1,2. At the interface of the dimer units, a channel is formed, which provides two binding sites for small lipophilic substances. The binding sites possess identical molecular organization and each is composed of three main elements, including a hydrophilic centre, a hydrophobic patch and a group of charged amino acid residues, which represent the entrance of the binding cavity 2,3. The natural endogenous ligands of these binding sites are the thyroid hormones triiodothyronine (T3) and thyroxine (T4) 4,5 and their binding is accomplished by the interaction of the iodine atoms with amino acid residues within the centre and the patch of the binding sites 3. In blood, TTR facilitates the transport of approximately 20% of circulating thyroid hormones, while in the cerebrospinal fluid, TTR is the major transport protein of thyroid hormones 3.

Thyroid hormones act intracellularly as transcription factors *via* binding to the thyroid hormone receptor. In this context, they are involved in the regulation of numerous physiological processes such as growth and development as well as the overall metabolic rate 5. To avoid detrimental metabolic consequences because of an imbalance in thyroid hormone homoeostasis, the cellular availability of thyroid hormones is strictly regulated. This is accomplished among other things by their interaction with the specific transport proteins. The dissociation of thyroid hormones from these proteins is the rate-determining step of their cellular uptake. In this context, particularly TTR is regarded as a potent modulator of thyroid hormone activity. As the affinity of TTR for thyroid hormones corresponds to their physiological concentration in the nanomolar range, the interaction of TTR with thyroid hormones represent a dynamic regulator of their cellular availability 4–7.

However, the affinity of TTR for thyroid hormones can be influenced by several factors. Drugs and toxins with thyroid hormone-like structure can displace thyroid hormones from their TTR-binding sites 4,8. In addition, several point mutations of TTR have also been associated with either increased or reduced affinity for thyroid hormones 3. Nevertheless, the effects of the most frequent alterations of the TTR molecules, namely oxidative post-translational modifications (PTM), on thyroid hormone binding have been mainly neglected so far. PTM of TTR predominantly affect the cysteine residue at position 10 (Cys_10_) of the polypeptide chain, which is not involved in any intra- or inter-protein disulphide bond 9. However, Cys_10_ is known to be susceptible to several oxidative modifications such as S-sulphonation and formation of mixed disuphides with cysteine, cysteinylglycine and glutathione 9–12 and up to 80% of the TTR molecules are affected by these oxidative modifications in blood and cerebrospinal fluid 12–15.

As the Cys_10_ residues are part of the thyroid hormone-binding channels within the TTR tetramer 7, we hypothesize that PTM of Cys_10_ reveal great potential to interfere with the thyroid hormone binding. To evaluate this assumption, we analysed the consequences of S-sulphonation, S-cysteinylation, S-cysteinylglycination and S-glutathionylation of TTR on thyroid hormone binding *in silico*. Furthermore, we aimed to evaluate the importance of PTM of TTR for the regulation of thyroid hormone homoeostasis *in vivo*. For this purpose, we applied a combination of immunoprecipitation and matrix-assisted laser desorption/ionization-time of flight-mass spectrometry (MALDI-TOF-MS) to analyse the PTM pattern of TTR in serum of patients with thyroid disease that reveal a dysregulation of thyroid hormone metabolism 16,17, and compared these results with the patterns obtained from serum of healthy controls. Finally, we also evaluated the relation of PTM of TTR with tetramer stability *in vivo*, because several *in vitro* experiments indicated a reduced stability of TTR tetramers induced by PTM 18–20.

## Materials and methods

### Molecular modelling

Molecular docking and energy minimization experiments were performed with the Molecular Operating Environment (MOE) molecular modelling program 2012.10 (Chemical Computing Group Inc., Montreal, QC, Canada) and Yasara 13.9.8 21. X-ray structures from the Protein Data Bank (PDB; IDs: 3KGT, 1FHN, 1ETB, 3KGS, 3M1O, 1THA and 4FI8) were used as templates for TTR 22. For homology modelling, the TTR sequence from Uniprot (ID: P02766) was utilized. Structures were prepared with the Protonate3D application of the MOE program 2012.10 23. The homology modelling macro of Yasara was utilized and the consensus models were performed with the MOE program 2012.10 and Yasara 13.4.21. Refinement of consensus models was done by molecular dynamics (MD) simulation. The stereochemistry quality aspects of the resulting models were checked *via* the MOE program 2012.10. The initial structure of 3,3′-diiodothyronine (T2) was transferred from the PDB (structure ID: 1THA) into the homology modelling. The T3 was built in the model from the T2 TTR tetramer and was refined. The optimal complex for each substituent and receptor was then subjected to MD using Yasara dynamics Yamber force field 24. A simulation cell was constructed around the TTR model (2*7.5 Å larger than the model) with a 7.9 Å real space cut-off for the electrostatic force calculated *via* the Particle Mesh Ewald method. The pKa values of the ionizable groups were predicted and protonation states were assigned based on pH 7.04 (temperature = 298 K, density = 0.997). The cell was filled with water and the Yamber electrostatic potential was evaluated at all water molecules, the one with the lowest or highest potential was turned into sodium or chloride counter ions until the cell was neutral. A short steepest descent minimization was done to remove severe bumps followed by simulated annealing minimizations at 298 K. The steered molecular dynamics (SMD) simulations were done with an amber 03 force field at 298 K and 0.9% NaCl on pH 7.0 in the simulation cell. For further analysis, simulation snapshots were captured from the beginning of the simulation and continuing with a total of 500 simulated annealing steps.

The available X-ray structures of TTR partially lack the N-terminal amino acid residues. Therefore, it was assured by the use of Yasara/MOE that the generated model included the amino acid residues Gly_6_-Glu_7_-Ser_8_-Lys_9_-Cys_10_ to consider the effect of modifications of Cys_10_ on N-terminal dynamics. The SMD transjectory was analysed with Vega ZZ V3.02 (freeware from Drug Design Laboratory, Milano, Italy) 25.

**Fig 2:**
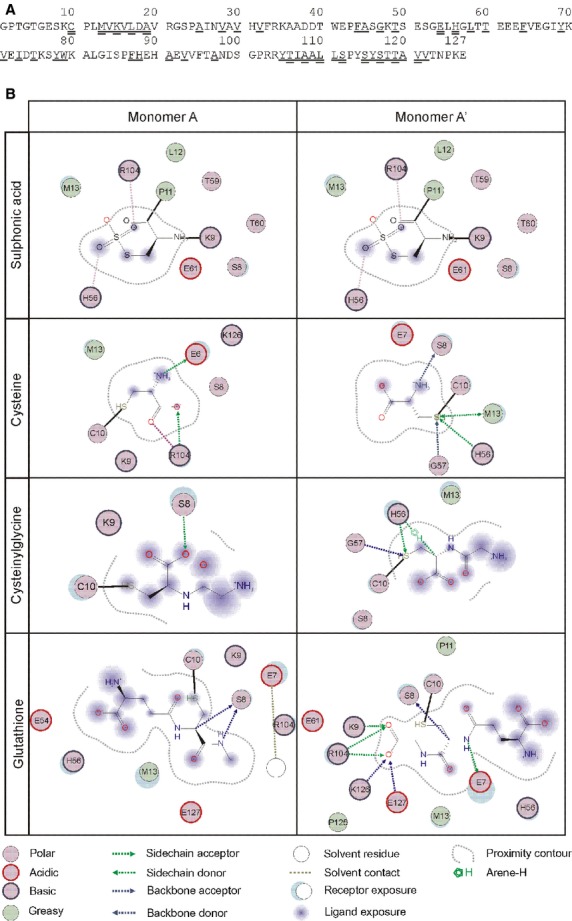
Amino acid sequence of human transthyretin polypeptide chain and its interaction with different substituents of Cys_10_. Amino acid sequence in single letter code is shown in A and was adapted from 7. Single underlined amino acids are residues in the core of the thyroid hormone-binding site and double underlined amino acid are residues within the central channel. Modification of Cys_10_ influences binding characteristics of the tertiary structure and the magnitude of the effects depend on the chemotype of the substituent as well as the position of the modified monomer within the tetrameric structure (B).

The TTR tetramer provides two thyroid hormone-binding sites and their entrances are formed by the monomers A and A' as well as the monomers B and B' (Fig.1), 2,3. For the steered dynamic calculation, the TTR model was used either unmodified or substituted on the Cys_10_ of monomer A and monomer A' (covering one thyroid hormone-binding site) by sulphonic acid, cysteine, cysteinylglycine and glutathione respectively. To evaluate the effect of PTM on T3 binding, the monomers B and B' within the TTR tetramer always remained unmodified and were used as reference (Table1).

**Table 1:** Changes in theoretical ejection times (ET) of triiodothyronine from transthyretin tetramer because of post-translational modifications (PTMs) of cysteine residue in position 10 (Cys_10_) determined by molecular modelling*

PTM Cys_10_ in thyroid hormone-binding cavity AA'			PTM Cys_10_ in thyroid hormone-binding cavity BB'			
Monomer A	Monomer A'	ET_A_	Monomer B	Monomer B'	ET_B_	ET_A_/ET_B_
None	None	30 ps	None		22 ps	1.36
S-sulphonation	None	30 ps	None		22 ps	1.36
S-sulphonation	S-sulphonation	38 ps	None		22 ps	1.73
S-cysteinylation	None	30 ps	None		45 ps	0.67
S-cysteinylation	S-cysteinylation	22 ps	None		22 ps	1.00
S-cysteinylglycination	None	37 ps	None		25 ps	1.48
S-cysteinylglycination	S-cysteinylglycination	83 ps	None		30 ps	2.77
S-glutathionylation	None	22 ps	None		22 ps	1.00
S-glutathionylation	S-glutathionylation	120 ps	None		22 ps	5.45

* TTR tetramer is composed of four identical subunits. Two subunits (A and B as well as A' and B') associate to form dimers (AB and A'B', respectively) and two dimers associate to form the tetramer. The thyroid hormone-binding cavities are located at the interface between the dimer units and their entrances are formed by the monomers AA' and BB' respectively 2,3. For molecular modelling, either one or both monomers of the thyroid hormone-binding cavity AA' were modified by sulphonic acid, cysteine, cysteinylglycine or glutathione at a time. The monomers of the thyroid-binding cavity BB' always remained unmodified. For detailed description of data generation, please see Material and Methods section.

Partial charges of PTM were considered. The time for ejection of T3 from the substituted TTR subunits A and A' was estimated based on the generated distance plots and compared to the data obtained for the unmodified subunits B and B'. Complete ejection of T3 was defined as a distance of 20 Å from amino acid residue Ala_108_, which is part of the T3-binding cavity and located within the central channel (Fig.2 and Data S1).

For the verification of computer- and program-specific bias, the calculation was repeated with another computer system. Calculations were performed with a computer system workstation with Dual Xenon Quad Cores (WinXP 32bit, Microsoft Corporation, Redmond, Washington, USA) and results were verified by the use of a computer system workstation with Intel I7 3930X Hexcore (Win7 64bit, Microsoft Corporation, Redmond, Washingtong, USA).

### Study population

A total of 80 hypothyroid (23 male and 57 female) and 18 hyperthyroid patients (5 male and 13 female) as well as 49 healthy, euthyroid individuals (30 male and 19 female) were recruited at the Department of Internal Medicine of the Acibadem University, Istanbul, Turkey, and were included in the study. Hypothyroidosis and hyperthyroidosis were initially diagnosed in all patients based on thyroid-stimulating hormone (TSH) serum concentration and/or thyroid hormone status. Hypothyroid patients were additionally classified as ‘incident’ (untreated; *n* = 21) or ‘treated with levothyroxine’ (*n* = 59). Hyperthyroid patients were all untreated.

Blood was drawn after an overnight fast during routine examination, immediately processed and the obtained serum was aliquoted and stored at −80°C until assayed. For all analyses, the samples were always kept at 4°C to avoid the introduction of artificial oxidative modifications. The study design was approved by the Medical Research Evaluation Ethical Committee of the Acibadem University.

### Isolation of TTR from human serum by immunoprecipitation and MALDI-TOF-MS analysis

Enrichment of TTR from serum by immunoprecipitation and subsequent MALDI-TOF-MS analysis was performed as previously described 13. For MALDI-TOF-MS analysis, 100 mM 2,5-dihydroxyacetophenone in 75% ethanol/20 mM dihydroxyammonium hydrogen citrate was used as matrix and calibration was accomplished by the use of an external calibration standard including insulin, cytochrome C, myoglobin and ubiquitin I (Bruker Daltonik GmbH, Bremen, Germany). Mass spectrometric analysis was accomplished with an Autoflex Speed MALDI-TOF mass spectrometer equipped with a neodymium-doped yttrium aluminium garnet laser (Bruker). The analyses were performed in positive ion and linear TOF mode with 19.5 kV acceleration voltage and a total of 1500 shots were collected per target spot in random walk using three spots per sample.

### Evaluation of MALDI-TOF mass spectra

Mass spectra were evaluated using flexAnalysis software (Bruker) and Excel (Microsoft Office 2007, Microsoft Corporation, Redmond, Washingtong, USA) as previously described 13. All spectra were screened for the presence of unmodified TTR (molecular weight 13,761 Da, 26) as well as the four oxidized TTR variants S-sulphonated TTR (sulphTTR), S-cysteinylated TTR (cysTTR), S-cysteinylglycinated TTR (cysglyTTR) and S-glutathionylated TTR (glutTTR), which are characterized by a mass shift of approximately +80 Da, +120 Da, +177 Da and +307 Da respectively 10–12. In addition, Gly-TTR, which is characterized by a mass shift of approximately −46 Da, was detected 11. The intensities of the six TTR variants Gly-TTR, unmodified TTR, sulphTTR, cysTTR, cysglyTTR and glutTTR were summed and regarded as the total intensity of TTR for each MALDI-TOF mass spectrum. The intensity of each TTR variant was then expressed as per cent of the total intensity for each spectrum and is referred to as relative intensity. The relative intensities of the TTR variants were used for the final data evaluation.

### Determination of TTR serum concentration

Transthyretin serum concentration was determined by a non-commercial ELISA as previously described, but using an in-house biotinylated rabbit-anti-human prealbumin antibody (DAKO, Hamburg, Germany) diluted 1:4000 in combination with horseradish peroxidise-labelled streptavidin (Dianova, Hamburg, Germany) diluted 1:6500 for detection 27.

### Evaluation of TTR tetramer stability by semi-native immunoblotting

The TTR tetramer is stable against most alkali and acid solutions as well as denaturating agents. However, the tetramer is sensitive to reducing agents and heating 1. Therefore, the percentage of tetrameric and monomeric TTR can be estimated by immunoblotting after performing a semi-native PAGE using SDS, but avoiding reducing agents as well as heating of the samples as demonstrated by Kingsbury *et al*. 19. For this purpose, mini-gels were prepared according to Laemmli, which were composed of 12% separation gels and 4% stacking gels 28. The serum samples were diluted 1:50 using a sample buffer containing 125 mmol/l Tris, 2% SDS, 20% glycerol and 0.1% bromophenol blue (pH 6.8). One serum sample was treated with a sample buffer additionally included 2% 2-mercaptoehtanol and heated to 95°C for 5 min. This procedure induced the dissociation of the tetramer and therefore TTR was predominantly present in its monomeric form in this sample and served as assay control. Samples were applied to the gels with 10 μl per slot. Electrophoresis was performed with 50 mA per gel using a commercial Tris/glycine/SDS running buffer (Bio-Rad, Munich, Germany).

Immunoblotting was performed as previously described using a polyclonal rabbit-anti-human prealbumin as primary antibody (DAKO) and HRP-conjugated polyclonal swine-anti-rabbit immunoglobulin (DAKO) as secondary antibody 29. The relative abundance of tetrameric and monomeric TTR was determined by densitometry using a Universal Hood II (Bio-Rad) for image detection and QuantityOne® version 4.5.2 (Bio-Rad) for data evaluation.

### Determination of total antioxidant capacity in serum

Total antioxidant capacity (TAC) was determined by the ferric reducing ability of plasma assay according to Benzie and Strain using ascorbic acid for calibration (50–1000 μmol/l) 30. Briefly, 10 μl of serum, calibrant and blank (water) were applied to a 96-well microtitre plate, respectively, and mixed with 150 μl of reactant solution, which was prepared by mixing equal amounts of 20 mM iron(III)-chloride (Sigma-Aldrich, Munich, Germany) in 250 mM acetic acid (pH 3.6) and 10 mM 2,4,6-tris(2-pyridyl)-s-triazine (Sigma-Aldrich) dissolved in 40 mM hydrochloric acid. The absorbance was measured at 595 nm after incubation for 6 min. in the dark. All analyses were performed in triplicates and the TAC is given as μmol/l ascorbic acid equivalents.

### Statistical analysis

Statistical analyses were accomplished by the use of IBM SPSS Statistics version 21. Results are expressed as mean ± SD or median (minimum-maximum) as appropriate. Normal distribution was tested by the use of Kolmogorov–Smirnov test. Non-normally distributed data were ln-transformed. Single factor anova with least significant difference as *post hoc* analysis was used for data evaluation.

To evaluate associations among the parameters, Pearson correlation analysis was performed. *P* < 0.05 (two-sided) was considered to be statistically significant.

## Results

### Building of TTR models for *in silico* analyses by molecular modelling

For molecular modelling, X-ray structures of TTR from the PDB were used as templates and the resulting TTR tetramer model including T3 is shown in Figure1 with each monomer in a different colour.

To analyse the effect of PTM on T3 binding, either one or both monomers of the thyroid hormone-binding site AA' were modified by one of the substituents sulphuric acid, cysteine, cysteinylglycine or glutathione. The thyroid hormone-binding site BB' always remained unmodified.

The left column of Figure1 exemplarily depicts the TTR tetramer with PTM of both monomers constituting one thyroid hormone entrance site by cysteinylglycine. The figure illustrates that the substituents tends to point towards the entrance of the binding channel and therefore may represent a sterical hindrance for T3 binding. In addition, the length of the thyroid hormone entrance channel is modulated by PTM with cysteinylglycine and glutathione, respectively (Data S2).

### Interaction of Cys_10_ substituents with the amino acids of the TTR polypeptide chain

The amino acid sequence of TTR is shown in Figure2A 7. Molecular interactions of Cys_10_ substituents and amino acids of the TTR polypeptide chain according the generated model for *in silico* analysis are shown in Figure2B and interaction characteristics of the monomers A and A' were considered.

By this approach, we were able to demonstrate that S-sulphonation of Cys_10_ did not result in formation of any considerable intramolecular interactions neither for monomer A nor for monomer A'. However, the formation of mixed disulphides resulted in reorganization of intramolecular interactions in a substituent- and monomer-specific manner. In monomer A, the substituents cysteine, cysteinylglycine and glutathione mainly interacted with other amino acids of the TTR N-terminus (Glu_6_ and Ser_8_), which are not directly involved in thyroid hormone binding. In addition, cysteine interacted with Arg_104_, which is also not directly involved in thyroid hormone binding, but located close to the core of the binding site. In contrast, for monomer A', more distinct reorganization processes were determined. Cysteine and cysteinylglycine entered only a limited number of interactions. However, these involved amino acids, which are part of the central thyroid hormone-binding channel (Met_13_ and His_56_). Instead, glutathione liaised with several amino acids within the TTR polypeptide chain. Although none of them is directly involved in thyroid hormone binding, their localization at the centre (Arg_104_), the N-terminus (Glu_7_, Ser_8_ and Lys_9_) as well as the C-terminus (Lys_126_ and Glu_127_) of the TTR monomer may influence the overall molecular structure of the TTR tetramer.

### Effect of Cys_10_ modifications on T3 binding estimated by ejection times

The effects of PTM of TTR on binding characteristics of T3 were evaluated by the estimation of T3 ejection times, which were calculated for the substituted (AA') as well as the unsubstituted binding site (BB') of one TTR tetramer and are presented in Table1.

In the completely unmodified TTR tetramer model, the observed ejection times slightly differed between the thyroid hormone cavities, indicating a higher affinity of binding cavity AA' for T3 (30 ps) in comparison to binding cavity BB' (22 ps). Introduction of any PTM resulted in changes of these ejection times. However, the effect substantially depended on the chemotype as well as the number of the introduced substituents.

In detail, modification of solely monomer A did not result in considerable effects on ejection times independent from the substituent used. The ratio of ejection times between the binding cavities (ET_A_/ET_B_) remained mostly unchanged under these conditions and only the substituents cysteine and glutathione resulted in minor changes (Table1).

In contrast, modification of both monomers, A and A', induced substituent-specific modification of T3 ejections times. In general, modification of both monomers resulted in an increase in ejection times of T3 from binding cavity AA'. However, this effect was only marginal for the small substituent sulphonic acid (38 ps), but substantial for the substituents cysteinylglycine (83 ps) and glutathione (120 ps) and resulted in a dramatic increase in the ejection time ratio ET_A_/ET_B_ between the binding cavities (2.77 and 5.45, respectively; Table1). The slowing of ejection time by double-modification with cysteinylglycine or glutathione is thereby most likely attributed to the elongation and splitting of the thyroid hormone-binding cavity's entrance into two tunnels by both substituents in contrast to the unmodified and S-sulphonated TTR (Fig.1 and Data S2).

An exception of the general tendency that with increasing molecular weight of the substituent the ejection times increase, was the modification of both monomers with cysteine. In contrast to the other substituents, modification with cysteine had only minor effects and by trend rather decreased the ejection time of T3 (Table1).

### Characterization of study population

Characteristics of the study population are presented in Table2. A total of 147 individuals were recruited and classified according to their thyroid status as euthyroid (*n* = 49), hyperthyroid (*n* = 18) or hypothyroid (*n* = 80). The hypothyroid patients were additionally subdivided as ‘incident’ (*n* = 21) or ‘treated with levothyroxine' (*n* = 59). The mean age of the study population was 47 ± 16 years with no differences between study groups. Because TSH serum concentration is closely related to thyroid function, significant differences were obvious between the study groups. Briefly, TSH serum concentration was highest in incident thyroid patients [6.17 mIU/l (4.39–15.0)] and lowest in hyperthyroid patients [0.01 mIU/l (0.00–0.27)] in comparison to each other as well as to euthyroid patients [1.76 mIU/l (0.01–3.13), *P* < 0.001 for both]. Treatment with levothyroxine resulted in normalization of TSH serum concentration in most hypothyroid patients [4.39 mIU/l (0.00–92.9)] and was significantly higher than in hyperthyroid patients (*P* < 0.001) and lower than in incident hypothyroid patients (*P* < 0.001), but comparable to euthyroid individuals (*P* = 0.811). The TAC also did not differ among the study groups.

**Table 2:** Clinical characteristics, TTR concentration and relative quantity of monomeric TTR in serum of the study population*

			Hypothyroid	
Parameter	Euthyroid	Hyperthyroid	Incident	Levothyroxine treatment
N (m/f)	49 (30/19)	18 (5/13)	21 (0/21)	59 (23/36)
Age (years)	50 ± 19	45 ± 17	48 ± 13	45 ± 14
TSH (mIU/l)	1.76 (0.01–3.13)a	0.01 (0.00–0.27)b	6.17 (4.39–15.0)c	4.39 (0.00–92.7)a
TAC (μmol/l ascorbic acid equivalents)	438 ± 118	372 ± 77.4	400 ± 59.6	397 ± 100
TTR (μmol/l)	4.55 ± 1.57	3.83 ± 1.69	4.37 ± 1.33	4.34 ± 1.51
Monomeric TTR (%)	3.01 (0.41–17.9)	2.44 (1.17–7.00)	2.71 (1.32–16.3)	2.83 (1.08–15.5)

m/f, male/female; TAC, total antioxidant capacity; TSH, thyroid-stimulating hormone; TTR, transthyretin.

* Data are presented as mean ± SD and median (minimum-maximum) as appropriate. Different superscripts (^a,b,c^) indicate significant differences between the groups with *P* <^ ^0.050 (two-tailed).

### TTR serum concentration and post-translational modifications of TTR

The average TTR serum concentration was 3.85 ± 2.95 μmol/l and was neither affected by hyperthyroidism nor by hypothyroidism (Table2).

**Fig 3:**
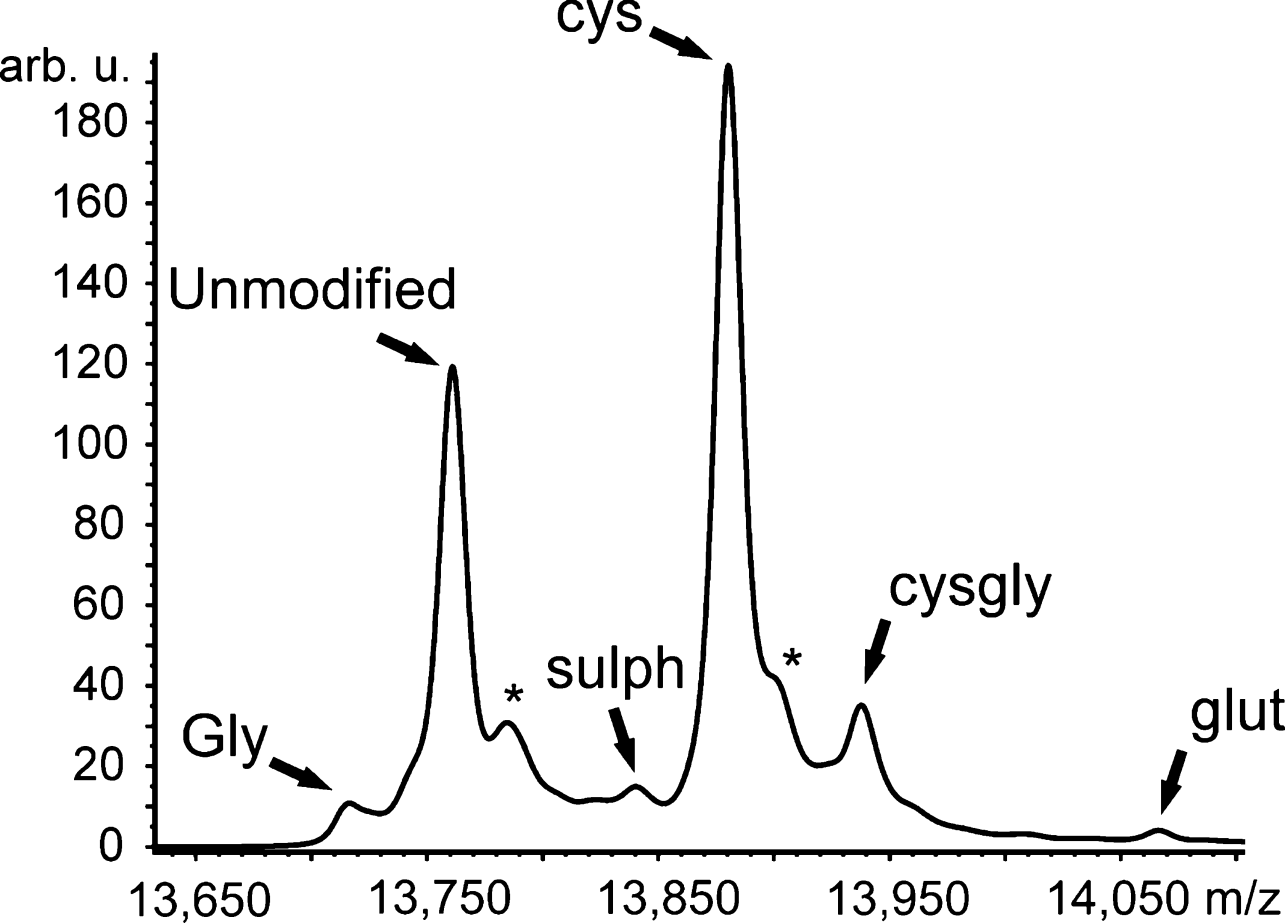
MALDI-TOF mass spectrum of transthyretin (TTR) after immunoprecipitation from serum. According to the molecular weight, the TTR variants were assigned as follows: TTR in which the cysteine side chain is chemically modified to form glycine (Gly, 13,718 Da), unmodified TTR (13,762 Da), sulphonated TTR (sulph, 13,841 Da), cysteinylated (cys, 13,881 Da), cysteinylglycinated TTR (cysgly, 13,938 Da), and glutathionylated TTR (glut, 14,067 Da) 11,12,26. The asterisk indicates adducts with sodium (+22 Da).

A representative MALDI-TOF mass spectrum of TTR is shown in Figure3. The molecular weights of the detected TTR variants were in accordance with previous reports and were as follows: 13,762 ± 2 Da for unmodified TTR, 13,842 ± 2 Da for sulphTTR, 13,882 ± 2 Da for cysTTR, 13,939 ± 2 Da for cysglyTTR and 14,067 ± 5 Da for glutTTR 10–12. In addition, a TTR variant with a molecular weight of 13,718 ± 2 Da was detected and assigned as Gly-TTR 11,12.

**Fig 4:**
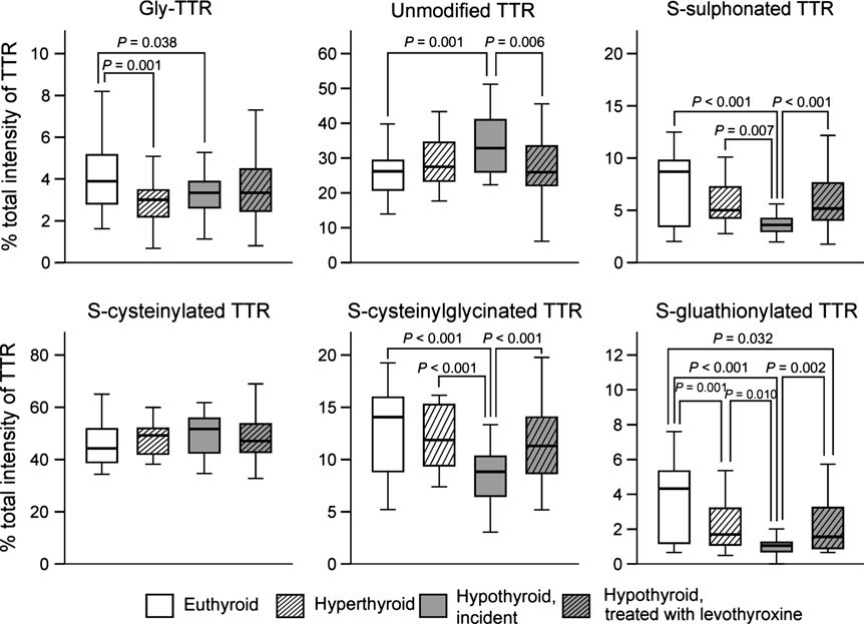
Relative amounts (%) of unmodified TTR and TTR post-translationally modified at Cys_10_ in serum of euthyroid patients as well as incident hyperthyroid patients, incident hypothyroid patients and hypothyroid patients with levothyroxine therapy determined by immunoprecipitation and subsequent MALDI-TOF-MS. Abbreviations used: Gly-TTR, TTR in which the cysteine side chain is chemically modified to form glycine; MALDI-TOF-MS, matrix-assisted laser desorption ionization-time of flight-mass spectrometry; TTR, transthyretin.

The most prominent modification of TTR *in vivo* was S-cysteinylation, which affected approximately 50% of all TTR molecules with no differences among the groups (Figs3 and 4). However, the relative amounts of the other PTM of TTR revealed thyroid function specificity as shown in Figure4. The most considerable differences in this context were detected between incident hypothyroid and euthyroid patients. Patients with incident hypothyroidism were characterized by elevated relative amounts of unmodified TTR [32.8% (22.3–51.2) *versus* 26.2% (14.0–44.0); *P* = 0.001] and reduced relative amounts of Gly-TTR [3.34% (1.13–6.63) *versus* 3.89% (1.62–8.20), *P* = 0.038], sulphTTR [3.61% (1.98–5.61) *versus* 8.70% (2.03–12.5), *P* = 0.001], cysglyTTR [8.82% (3.05–13.3) *versus* 14.1% (5.22–19.3), *P* < 0.001] and glutTTR [1.05% (0.00–2.16) *versus* 4.32 (0.65–7.61), *P* < 0.001]. However, these differences were mainly normalized in hypothyroid patients treated with levothyroxine and only relative amounts of glutTTR remained reduced [1.56% (0.66–10.9), *P* = 0.032] in comparison to euthyroid patients. In case of hyperthyroidism, only minor differences in PTM pattern of TTR were detectable in comparison to euthyroid patients. In fact, only relative amounts of Gly-TTR [5.09% (1.13–6.63); *P* = 0.001] and glutTTR [1.68% (0.49–5.36); *P* = 0.007] were significantly reduced in this subgroup of patients.

### Evaluation of TTR tetramer stability

**Fig 5:**
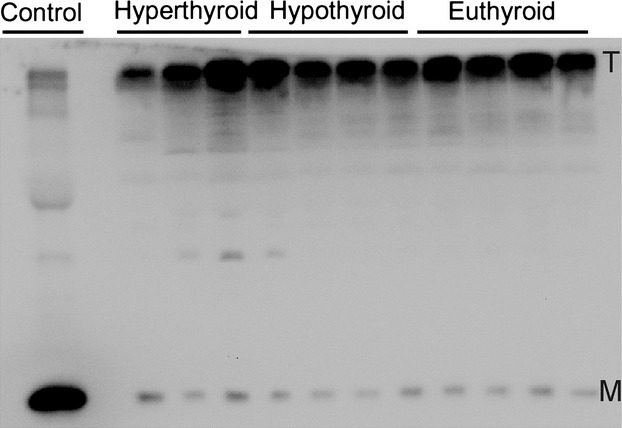
Discrimination of monomeric (M) and tetrameric (T) transthyretin (TTR) in serum of patients with hyperthyroidism and patients with hypothyroid as well as euthyroid patients determined by immunoblotting under semi-denaturating conditions. Samples were diluted 1:50 with a sample buffer containing SDS but not 2-mercaptoehtanol and were not heated. The control was a serum sample diluted 1:50 using a sample buffer with SDS and 2-mercaptoethanol and which was heated to 95°C for 5 min. Amounts of monomeric and tetrameric TTR were determined by densitometry.

Transthyretin is secreted as a tetramer, but its stability is known to be influenced among other things by PTM 9,18,19. Therefore, TTR tetramer stability *in vivo* was analysed by semi-native TTR immunoblotting and a representative immunoblot is shown in Figure5. One serum sample served as control and was analysed under denaturating conditions, hence containing primarily monomeric TTR. Densitometric analyses of the immunoblots revealed that only approximately 2–3% of TTR were present as monomers under semi-native conditions in all analysed samples with no differences among the groups (Table2).

### Correlation of PTM of TTR with TAC and tetramer stability

Due to the oxidative nature of the most PTM of TTR, their association with TAC *in vivo* was analysed by Pearson correlation analysis as shown in Table3. TAC was associated with relative amounts of all TTR variants, except Gly-TTR and cysTTR. In this context, the TAC was inversely correlated with the relative amounts of unmodified TTR (Pearson correlation coefficient −0.501, *P* < 0.001) and positively associated with the relative amounts of sulphTTR (Pearson correlation coefficient 0.447, *P* < 0.001), cysglyTTR (Pearson correlation coefficient 0.305, *P* = 0.002) and glutTTR (Pearson correlation coefficient 0.447, *P* < 0.001).

**Table 3:** Correlation analysis of PTM of TTR, TAC and amount of monomeric TTR*

	TAC	Gly-TTR	Unmodified TTR	sulphTTR	cysTTR	cysglyTTR	glutTTR
Monomeric TTR (%)	**−0.217** (*P* = 0.047)	−0.020 (*P* = 0.824)	0.131 (*P* = 0.145)	0.018 (*P* = 0.838)	−0.169 (*P* = 0.060)	−0.019 (*P* = 0.829)	0.000 (*P* = 0.996)
TAC		0.190 (*P* = 0.055)	−**0.501** (*P* < 0.001)	**0.447** (*P* < 0.001)	0.054 (*P* = 0.591)	**0.305** (*P* = 0.002)	**0.439** (*P* < 0.001)

Gly-TTR, TTR in which the cysteine side chain is chemically modified to form glycine; cysTTR, S-cysteinylated TTR; cysglyTTR, S-cysteinylglycinated TTR; glutTTR, S-glutathionylated TTR; sulphTTR, S-sulphonated TTR; TAC, total antioxidant capacity in μmol/l ascorbic acid equivalents; TTR, transthyretin.

* Results of Pearson correlation analysis. Statistically significant correlations are highlighted in bold.

In addition, the effect of PTM of TTR on the tetramer stability was evaluated and the results are also presented in Table3. The amount of monomeric TTR in serum did not correlate with the appearance of any PTM of TTR. However, this parameter was marginally inversely associated with the TAC (Pearson correlation coefficient −0.217, *P* = 0.047).

## Discussion

Cysteine residues are often critical catalytic and structural elements, which are important for protein function and processing 31. In addition, they are prone to oxidative modifications that can substantially affect protein characteristics 32. In this context, we have demonstrated in this study by molecular modelling that PTMs of Cys_10_ within the TTR molecule interfere with its thyroid hormone binding properties. Furthermore, we provided evidence that the PTM pattern of TTR is associated with thyroid function *in vivo*, probably indicating its implication in fine-tuning of thyroid hormone homoeostasis.

Transthyretin is constituted as a homotetramer, providing two binding sites for thyroid hormones and the binding of thyroid hormones is facilitated by the interaction of the iodine atoms with amino acid residues within the centre and the patch of the binding sites 3. Because of the differing amount of incorporated iodine atoms, the affinity of TTR for T3 and T4 differs by an order of magnitude with approximately 1 × 10^−7^ M for T3 and 7 × 10^−7^ M for T4 5.

Although, T4 is the predominant thyroid hormone in the circulation, the molecular modelling simulations of this study were focused on the effect of PTM of TTR on binding of T3. This is mainly attributed to the fact that T3 is commonly regarded as the active thyroid hormone, responsible for most of the thyroid hormone functions, and is also more rapidly taken up by the target cells than T4 33. In addition, T3 serum concentration is more sensitive to exogenous factors such as food intake, exercise and severe illness 5,34 and requires considerably more precise regulation to maintain the hormone homoeostasis. Nevertheless, because of the highly similar structure and binding characteristics of T3 and T4, we assume comparable *in silico* results for T4 3,5.

In this context, the molecular modelling scenarios designed herein demonstrated that for the TTR tetramer without any modification of Cys_10_, the stability of T3 binding, estimated by binding site-specific ejections times, differed between the binding cavities (Table1). This is in accordance with previous reports and mainly based on a marginal asymmetry of the TTR tetramer, resulting in a slight difference in the size of the thyroid hormone-binding sites. Therefore, one binding site is preferentially occupied by thyroid hormones under physiological conditions 7. The introduction of PTM of Cys_10_ affected the stability of T3 binding in the modelled scenarios beyond this constitutional difference. In principle, the stability of T3 binding was increased with increasing number and molecular weight of the substituents with most pronounced alterations for modifications with cysteinylglycine and glutathione (Table1). However, an exception of this trend was the cysteinylation, which rather resulted in a slight reduction in T3 binding stability.

An influence of Cys_10_ modifications on binding of thyroid hormones to TTR has been suggested before, but was not analysed in detail yet 20. Based on the results of this study, several mechanisms are conceivable by which PTM may affect the interaction of TTR and T3 in a chemotype-specific manner. Firstly, as Cys_10_ is part of the thyroid hormone-binding channel 7, its modification may introduce a sterical hindrance for T3 binding. This hypothesis is supported by the molecular modelling simulations of this study, which indicate that the substituents are directed towards the thyroid hormone-binding site in the TTR tetramer and tend to elongate the entrance channel (Fig.1 and Data S2).

Secondly, the substituents interact differentially and site-specific with amino acids of the TTR subunits (Fig.2). In this context, interactions with amino acids directly involved in thyroid hormone binding as well as with amino acids away from the thyroid hormone-binding sites are established. As it is known from previous studies, which analysed the effect of single amino acid changes, that even substitutions away from the thyroid hormone-binding site can substantially affect the affinity for the ligands 3, it seems to be rational that PTMs of TTR also induce conformational changes, which result in narrowing or widening of the thyroid hormone-binding cavity or -binding channel and subsequently cause changes in T3-binding characteristics. This rather global approach would also explain the effects of PTMs on T3 binding of the non-modified binding site as well as the amplification of the effect by introduction of a second PTM, but also the diverse effects according the chemotype of the substituents (Table1).

Finally, Cys_10_ is also a part of the N-terminal tail of the TTR polypeptide chain, which is supposed to be of substantial relevance for thyroid hormone binding. It has been demonstrated that length and amino acid composition of the N-terminus determines the affinity of TTR for T3 and T4. Moreover, it has been suggested that the N-terminal tail of TTR moves freely in solution and is involved in thyroid hormone binding by interacting with the entrance of the binding sites 7. Based on this relation and the fact that according to this study, particularly the substituents cysteine, cysteinylglycine and glutathione interact with amino acids of the N-terminus (Fig.2), it may be speculated that the modifications of Cys_10_ affect the orientation and flexibility of the N-terminal tail in relation to the binding cavity's entrance and thereby manipulate thyroid hormone binding.

These complex associations determined by molecular modelling raised the question of the physiological relevance of the Cys_10_ modifications. Previous reports have revealed an association of PTM of TTR with cancer 15, medication 13 as well as nutritional status 29. The underlying mechanisms for this coherence are not completely understood and only marginally investigated. However, all these conditions are associated with complex metabolic changes, which in all likelihood involve thyroid hormones or affect their metabolism as central mediators of the body's homoeostasis 17,35. On the basis of this assumption, we assumed an association of PTM of TTR with the thyroid status, which could be confirmed in this study. We were able to demonstrate that particularly patients with untreated (incident) hypothyroidism revealed substantial changes in the PTM pattern of TTR in comparison to healthy, euthyroid patients (Fig.4). In this context, mainly S-sulphonation, S-cysteinylglycination and S-glutathionylation were associated with the thyroid function and supported the results of the molecular modelling analysis, which identified particularly these modifications of TTR as potent modulators of T3 binding (Table1 and Fig.4). In addition, we detected an association of S-sulphonation, S-cysteinylglycination and S-glutathionylation, but not S-cysteinylation, of TTR with the TAC in serum of the study cohort (Table3). As an imbalance of redox status is a frequent complication in thyroid disease 17,35 as well as other diseases such as cancer and malnutrition, this association may direct towards a possible biochemical mechanism to regulate the interaction of TTR and T3 (thyroid hormones). Based on the results of this study, it may be assumed that oxidative modification of TTR is a co-ordinated process to balance the thyroid hormone availability. Regulation of protein function by oxidative modifications of cysteine residues has been described before and affects different protein characteristics such as ligand binding and enzyme activity 36–40. Particularly, reversible modifications of cysteine have been suggested as potent regulatory switch of protein function, because these can be adjusted by a combination of several enzymatic systems such as glutathione/glutathione reductase 41. Furthermore, it has been demonstrated for several proteins that the chemotype of cysteine modification is also important for the regulation of protein function 40,42–44. The fact that in this study, predominantly reversible PTM of TTR (S-sulphonation, S-cysteinylation, S-cysteinylglycination, S-glutathionylation) were identified (Fig.3), may therefore support the hypothesis that PTM of TTR are directed modifications assisting the maintenance of thyroid hormone balance. The hypothesis of a condition-specific modification might be furthermore supported by the restoration of PTM pattern of TTR in hypothyroid patients treated with levothyroxine (Fig.4). Beyond this, PTM of TTR may also represent a mechanism to regulate the availability of thyroid hormones during embryonic development. Particularly during early stages of pregnancy, embryos depend on the supply with maternal thyroid hormones, which have to cross the placenta to interact with foetal thyroid receptors. In this context, the biological activity of thyroid hormones is regulated by the activity of transmembrane thyroid hormone transporters such as monocarboxylate transporter 8 and the activity of iodothyronine deiodinases that catalyse activation and degradation of thyroid hormones. However, particularly, the availability of thyroid hormones is decisive and determined by their release from the circulating transport proteins as well as the interaction of these proteins with the transmembrane transporters 45,46. Therefore, PTM of TTR may also be involved in the regulation of transplacental transport of thyroid hormones.

Nevertheless, previous reports suggested other mechanisms linking oxidative stress, thyroid hormone binding and oxidative PTM of TTR. Among these, the dissociation of the TTR tetramer with concomitant release of associated thyroid hormones is the most popular 1,9,20. In fact, several *in vitro* studies demonstrated that PTM of TTR promote the dissociation of the TTR tetramer 18,19. Therefore, we aimed to analyse this association in our study by quantifying the relative amount of monomeric TTR as an indirect indicator of tetramer dissociation *in vivo*. By this approach, we were not able to determine any association of PTM of TTR with the appearance of monomeric TTR neither by correlation analysis (Table3) nor by considering the thyroid status of the study population (Table2 and Fig.5). This is in conflict with the previous results mentioned above. However, these results were obtained by *in vitro* studies focusing on the amyloidogenicity of TTR and by application of unphysiological pH conditions (acidic or alkaline) to promote tetramer dissociation 18,19. Therefore, TTR tetramer dissociation may be excluded as a major mechanism for regulation of thyroid hormone balance under physiological conditions.

Finally, it has also to be considered that for the *in vivo* results of this study, no characterization of tetramer-specific PTM patterns were possible. The relative amounts of the analysed PTM of TTR by MALDI-TOF-MS represent their overall proportions and combinations of different PTMs for each individual TTR tetramer are possible. Conclusions concerning their effects on thyroid hormone binding hence would remain speculative, especially when taking into account the complex effects of PTM of Cys_10_ as indicated by molecular modelling.

In conclusion, the molecular modelling results of this study identified a complex influence of the TTR-T3 interaction by PTM of TTR and may be interpreted as a potential mechanism to maintain the thyroid hormone homoeostasis. This theory of co-ordinated oxidative modifications of TTR to adjust the thyroid hormone status to (micro-) environmental conditions is supported by the *in vivo* results, which revealed thyroid function-specific patterns of TTR modifications in serum. However, further research is needed to detect mechanisms, which determine degree, chemotype and site of TTR modification by analysing binding characteristics of thyroid hormones to differentially modified TTR in detail in various physiological and pathological conditions.

## Supporting Information

**Data S1** Interaction of triiodthyronine with amino acids of monomer A and monomer A', which are located within or close to the binding cavity (A) and sequential exit of triiodothyronine (T3) from the TTR tetramer (B).

**Data S2** Elongation of the thyroid hormone dissociation distance by splitting of the binding cavity's entrance into two tunnels by introduction of cysteinylglyicine and glutathione as post-translational modification of TTR subunits A and A'.

## References

[b1] Ingenbleek Y, Young V (1994). Transthyretin (prealbumin) in health and disease: nutritional implications. Annu Rev Nutr.

[b2] Blake CC, Geisow MJ, Oatley SJ (1978). Structure of prealbumin: secondary, tertiary and quaternary interactions determined by Fourier refinement at 1.8 A. J Mol Biol.

[b3] Hamilton JA, Benson MD (2001). Transthyretin: a review from a structural perspective. Cell Mol Life Sci.

[b4] Palha JA (2002). Transthyretin as a thyroid hormone carrier: function revisited. Clin Chem Lab Med.

[b5] Hulbert AJ (2000). Thyroid hormones and their effects: a new perspective. Biol Rev Camb Philos Soc.

[b6] Schreiber G, Richardson SJ (1997). The evolution of gene expression, structure and function of transthyretin. Comp Biochem Physiol B Biochem Mol Biol.

[b7] Prapunpoj P, Leelawatwattana L (2009). Evolutionary changes to transthyretin: structure-function relationships. FEBS J.

[b8] Munro SL, Lim CF, Hall JG (1989). Drug competition for thyroxine binding to transthyretin (prealbumin): comparison with effects on thyroxine-binding globulin. J Clin Endocrinol Metab.

[b9] Pettersson T, Carlstrom A, Jornvall H (1987). Different types of microheterogeneity of human thyroxine-binding prealbumin. Biochemistry.

[b10] Nedelkov D, Phillips DA, Tubbs KA (2007). Investigation of human protein variants and their frequency in the general population. Mol Cell Proteomics.

[b11] Kishikawa M, Sass JO, Sakura N (2002). The peak height ratio of S-sulfonated transthyretin and other oxidized isoforms as a marker for molybdenum cofactor deficiency, measured by electrospray ionization mass spectrometry. Biochim Biophys Acta.

[b12] Poulsen K, Bahl JM, Tanassi JT (2012). Characterization and stability of transthyretin isoforms in cerebrospinal fluid examined by immunoprecipitation and high-resolution mass spectrometry of intact protein. Methods.

[b13] Henze A, Raila J, Scholze A (2013). Does N-acetylcysteine modulate posttranslational modifications of transthyretin in hemodialysis patients?. Antioxid Redox Signal.

[b14] Frey SK, Spranger J, Henze A (2009). Factors that influence retinol-binding protein 4-transthyretin interaction are not altered in overweight subjects and overweight subjects with type 2 diabetes mellitus. Metabolism.

[b15] Fung ET, Yip TT, Lomas L (2005). Classification of cancer types by measuring variants of host response proteins using SELDI serum assays. Int J Cancer.

[b16] Johnson JL, Felicetta JV (1992). Hypothyroidism: a comprehensive review. J Am Acad Nurse Pract.

[b17] Franklyn JA, Boelaert K (2012). Thyrotoxicosis. Lancet.

[b18] Zhang Q, Kelly JW (2003). Cys10 mixed disulfides make transthyretin more amyloidogenic under mildly acidic conditions. Biochemistry.

[b19] Kingsbury JS, Laue TM, Klimtchuk ES (2008). The modulation of transthyretin tetramer stability by cysteine 10 adducts and the drug diflunisal. Direct analysis by fluorescence-detected analytical ultracentrifugation. J Biol Chem.

[b20] Pettersson TM, Carlstrom A, Ehrenberg A (1989). Transthyretin microheterogeneity and thyroxine binding are influenced by non-amino acid components and glutathione constituents. Biochem Biophys Res Commun.

[b21] Krieger E, Koraimann G, Vriend G (2002). Increasing the precision of comparative models with YASARA NOVA–a self-parameterizing force field. Proteins.

[b22] Berman HM, Westbrook J, Feng Z (2000). The protein data bank. Nucleic Acids Res.

[b23] Chun E, Thompson AA, Liu W (2012). Fusion partner toolchest for the stabilization and crystallization of G protein-coupled receptors. Structure.

[b24] Krieger E, Darden T, Nabuurs SB (2004). Making optimal use of empirical energy functions: force-field parameterization in crystal space. Proteins.

[b25] Pedretti A, Villa L, Vistoli G (2004). VEGA–an open platform to develop chemo-bio-informatics applications, using plug-in architecture and script programming. J Comput Aided Mol Des.

[b26] UniProt (2011). Ongoing and future developments at the Universal Protein Resource. Nucleic Acids Res.

[b27] Raila J, Henze A, Spranger J (2007). Microalbuminuria is a major determinant of elevated plasma retinol-binding protein 4 in type 2 diabetic patients. Kidney Int.

[b28] Laemmli UK (1970). Cleavage of structural proteins during the assembly of the head of bacteriophage T4. Nature.

[b29] Henze A, Rohn S, Gericke B (2008). Structural modifications of serum transthyretin in rats during protein-energy malnutrition. Rapid Commun Mass Spectrom.

[b30] Benzie IF, Strain JJ (1996). The ferric reducing ability of plasma (FRAP) as a measure of “antioxidant power”: the FRAP assay. Anal Biochem.

[b31] Bykova NV, Rampitsch C (2013). Modulating protein function through reversible oxidation: redox-mediated processes in plants revealed through proteomics. Proteomics.

[b32] Hill BG, Bhatnagar A (2012). Protein S-glutathiolation: redox-sensitive regulation of protein function. J Mol Cell Cardiol.

[b33] Hennemann G, Docter R, Friesema EC (2001). Plasma membrane transport of thyroid hormones and its role in thyroid hormone metabolism and bioavailability. Endocr Rev.

[b34] Ligtenberg JJ, Girbes AR, Beentjes JA (2001). Hormones in the critically ill patient: to intervene or not to intervene?. Intensive Care Med.

[b35] Larson J, Anderson EH, Koslawy M (2000). Thyroid disease: a review for primary care. J Am Acad Nurse Pract.

[b36] Nagumo K, Tanaka M, Chuang VT (2014). Cys34-cysteinylated human serum albumin is a sensitive plasma marker in oxidative stress-related chronic diseases. PLoS ONE.

[b37] Yang H, Lundback P, Ottosson L (2012). Redox modification of cysteine residues regulates the cytokine activity of high mobility group box-1 (HMGB1). Mol Med.

[b38] Ward NE, Chu F, O'Brian CA (2002). Regulation of protein kinase C isozyme activity by S-glutathiolation. Methods Enzymol.

[b39] Chu F, Ward NE, O'Brian CA (2003). PKC isozyme S-cysteinylation by cystine stimulates the pro-apoptotic isozyme PKC delta and inactivates the oncogenic isozyme PKC epsilon. Carcinogenesis.

[b40] Barrett WC, DeGnore JP, Konig S (1999). Regulation of PTP1B *via* glutathionylation of the active site cysteine 215. Biochemistry.

[b41] Paulsen CE, Carroll KS (2009). Orchestrating redox signaling networks through regulatory cysteine switches. ACS Chem Biol.

[b42] van Montfort RL, Congreve M, Tisi D (2003). Oxidation state of the active-site cysteine in protein tyrosine phosphatase 1B. Nature.

[b43] Chu F, Ward NE, O'Brian CA (2001). Potent inactivation of representative members of each PKC isozyme subfamily and PKD *via* S-thiolation by the tumor-promotion/progression antagonist glutathione but not by its precursor cysteine. Carcinogenesis.

[b44] Auclair JR, Johnson JL, Liu Q (2013). Post-translational modification by cysteine protects Cu/Zn-superoxide dismutase from oxidative damage. Biochemistry.

[b45] Nucera C, Muzzi P, Tiveron C (2010). Maternal thyroid hormones are transcriptionally active during embryo-foetal development: results from a novel transgenic mouse model. J Cell Mol Med.

[b46] Wirth EK, Schweizer U, Kohrle J (2014). Transport of thyroid hormone in brain. Front.

