# Supplementary material for: Post-translational modifications of transthyretin affect the triiodonine-binding potential
Source: J Cell Mol Med. 2014 Oct 14;19(2):359–70. doi: 10.1111/jcmm.12446 (PMC4407597; doi:10.1111/jcmm.12446)
Supplement: Supplementary file 2 [file jcmm0019-0359-sd2.docx]

Elongation of the thyroid hormone dissociation distance by splitting of the binding

cavity’s entrance into two tunnels by introduction of cysteinylglyicine and glutathione

as posttranslational modification of TTR subunits A and A’.*

| **PTM of Cys_10_ in monomers A and A’** | **Binding cavity channel** | **Channel length in Å from Ala_108_** |
| --- | --- | --- |
| unmodified | 1 | 14.23 |
|  | 2 | Non-existent |
| S-sulphonation | 1 | 14.23 |
|  | 2 | Non-existent |
| S-cysteinylglycination | 1 | 10.37 |
|  | 2 | 20.36 |
| S-glutathionylation | 1 | 15.24 (blocked) |
|  | 2 | 21.28 |

*TTR tetramer is composed of four identical subunits. Always two subunits (A and B as well as A’ and B’) associate to form dimers (AB and A’B’, respectively). Subsequently both dimer units associate to form the tetramer. The thyroid hormone binding cavities are located at the interface between the dimer units and their entrances are formed by the monomers AA’ and BB’, respectively [1, 2]. For molecular modelling either one or both monomers of the thyroid hormone binding cavity AA’ were posttranslaitonally modified at the Cys_10_ residue. In contrast, the monomers of the thyroid binding cavity BB’ always remained unmodified. The length of the original and split channels was calculated using Mole 2.13.9.6 software [3].

**References**

1 **Blake CC, Geisow MJ, Oatley SJ*, et al.*** Structure of prealbumin: secondary, tertiary and quaternary interactions determined by Fourier refinement at 1.8 A. *J Mol Biol.* 1978; 121: 339-56.

2 **Hamilton JA,Benson MD.** Transthyretin: a review from a structural perspective. *Cell Mol Life Sci.* 2001; 58: 1491-521.

3 **Sehnal D, Svobodova Varekova R, Berka K*, et al.*** MOLE 2.0: advanced approach for analysis of biomacromolecular channels. *J Cheminform.* 2013; 5: 39.
